# A comparative proteomic analysis provides insight into the molecular mechanism of bud break in longan

**DOI:** 10.1186/s12870-022-03868-3

**Published:** 2022-10-12

**Authors:** Dengwei Jue, Liqin Liu, Xuelian Sang, Shengyou Shi

**Affiliations:** 1 Chongqing Key Laboratory of Economic Plant Biotechnology, Collaborative Innovation Center of Special Plant Industry in Chongqing, Chongqing Engineering Research Center for Special Plant Seedling, Institute of Special Plants, Chongqing University of Arts and Sciences, 402160 Yongchuan, China; 2 Key Laboratory of Horticulture Science for Southern Mountains Regions of Ministry of Education, College of Horticulture and Landscape Architecture, Southwest University, 400715 Beibei, Chongqing, China; 3 Key Laboratory of Tropical Fruit Biology (Ministry of Agriculture), South Subtropical Crops Research Institute, Chinese Academy of Tropical Agricultural Sciences, 524091 Zhanjiang, China

**Keywords:** Longan, Flower bud break, iTRAQ, Carbohydrate, Photosynthesis

## Abstract

**Background:**

The timing of bud break is very important for the flowering and fruiting of longan. To obtain new insights into the underlying regulatory mechanism of bud break in longan, a comparative analysis was conducted in three flower induction stages of two longan varieties with opposite flowering phenotypes by using isobaric tags for relative and absolute quantification (iTRAQ).

**Results:**

In total, 3180 unique proteins were identified in 18 samples, and 1101 differentially abundant proteins (DAPs) were identified. “SX” (“Shixia”), a common longan cultivated variety that needs an appropriate period of low temperatures to accumulate energy and nutrients for flower induction, had a strong primary inflorescence, had a strong axillary inflorescence, and contained high contents of sugars, and most DAPs during the bud break process were enriched in assimilates and energy metabolism. Combined with our previous transcriptome data, it was observed that sucrose synthase 6 (SS6) and granule-bound starch synthase 1 (GBSSI) might be the key DAPs for “SX” bud break. Compared to those of “SX”, the primary inflorescence, axillary inflorescence, floral primordium, bract, and prophyll of “SJ” (“Sijimi”) were weaker. In addition, light, rather than a high sugar content or chilling duration, might act as the key signal for triggering bud break. In addition, catalase isozyme 1, an important enzyme in the redox cycle, and RuBisCO, a key enzyme in the Calvin cycle of photosynthetic carbon assimilation, might be the key DAPs for SJ bud break.

**Conclusion:**

Our results present a dynamic picture of the bud break of longan, not only revealing the temporal specific expression of key candidate genes and proteins but also providing a scientific basis for the genetic improvement of this fruit tree species.

**Supplementary Information:**

The online version contains supplementary material available at 10.1186/s12870-022-03868-3.

## Background

Longan (*Dimocarpus longan*) is a subtropical perennial crop, and it is best known for its nutritious fruit, which has a relatively high medicinal value [1]. A stable annual yield is the most important factor affecting the healthy development of the longan industry. However, the irregular flowering habit of longan as a biennial fruit tree often affects its production and leads to erratic yields [2]. There are many environmental conditions that can trigger the irregular flowering of longan, such as spring frost accompanied by flower damage and high temperature and moisture in winter, which causes flowering reversion [3, 4]. These adverse environmental conditions lead to longan bud break and flowering at inappropriate times. Therefore, understanding the genetic and physiological bases of bud break is of great importance to control longan fruit yield to establish regular annual cropping levels and to alleviate the production constraints associated with biennial bearing.

Bud dormancy is a biological characteristic and a necessary physiological process that enables plants to store more energy to survive for long periods under adverse conditions. There are three different stages that determine the quality of bud dormancy release, flowering, and fruit yield: (1) paradormancy, where growth inhibition arises from another part of the plant; (2) endodormancy (or true dormancy), which is triggered by internal factors; and (3) ecodormancy, which is controlled by environmental factors [5, 6]. In the context of global warming, knowledge of the physiological, biochemical, and molecular bases of bud break in perennial fruit trees is of crucial importance because the timing of bud break directly affects flowering quality and uniformity [7, 8].

In recent decades, many studies have been conducted on this issue, and many factors have been identified in temperate and boreal trees [9, 10], with winter chilling being the key environmental factor that controls their phenology. For plants with a winter annual life history, sufficient chill accumulation may be a critical step for bud break, and after bud break takes place, a period of mild temperature is required for growth resumption [7]. Another environmental factor is photoperiods. Plants that are sensitive to photoperiods do not have to rely on warm temperatures alone, thus protecting them when freezing weather returns (Keskitalo et al., 2005). Interestingly, it has been found that some plants can remember their prior chill accumulation; for example, vernalized henbane plants were grown in noninductive photoperiods (where they cannot flower), and when they were exposed to inductive photoperiods, they flowered [11]. Carbohydrate metabolism plays an important role in the process of bud break [7, 12] by acting as the primary source of carbon and energy [13]. A dormant bud’s capacity to release is tightly linked to its supply of carbohydrates. During dormancy, carbohydrate dynamics are restricted to bud tissues, and a sugar deficit is the cause of growth cessation and bud dormancy. In response to winter conditions, carbohydrates in dormant buds are synthesized from the reserves accumulated during the growing season. After bud break, the carbohydrate uptake capacity of a bud increases with an increase in the expression and activity of plasma membrane transporters [14]. Many studies have shown that there is a link between changes in carbohydrate dynamics and changes in dormancy status. For example, during the onset of dormancy in sweet cherry, starch is degraded into soluble sugars, and an increase in starch occurs before budburst [15]. With the overexpression of the *Arabidopsis* sucrose phosphate synthase gene, transgenic poplar lines showed earlier bud break than wild-type lines, which raises the possibility that enhanced sugar and/or starch reserves can promote accelerated bud break [16]. In addition to carbohydrate metabolism, redox signaling and phytohormone networks have been described to synergistically control growth, development, and differentiation, including bud break [17]. For example, in peach and sweet cherry, low-temperature stress was found to be closely linked to oxidative stress and to provoke ethylene biosynthesis, which is associated with dormancy release and bud break [18, 19].

In contrast to boreal and temperate trees, subtropical tree species grow in subtropical regions with short winters and relatively warm temperatures that rarely drop below 5 ℃, which is the typical minimum temperature needed for most temperate plants to meet their winter chilling requirement for bud break and flowering [10]. It seems that winter chilling and photoperiods may not be critical factors for bud break or flowering in subtropical tree species. In fact, some researchers believe that the growth, dormancy, and break of dormancy of subtropical and tropical fruit trees rely on more subtle changes in rainfall, temperature, and nutrient availability [20]. However, researchers recently found that chilling is also a driving force of rest (endodormancy) breaks in subtropical trees. However, the chilling requirement is lower in subtropical trees than in boreal and temperate trees [9, 10]. In addition, they also found that the chilling requirement is not the only factor that controls bud break in subtropical trees, as photoperiods can interact with chilling in some subtropical trees to precisely regulate budburst in a timely manner [10]. However, the roles of environmental factors in bud break and flowering are still largely unexplored.

According to the requirements of climate conditions and the variety of characteristics shown by their environments, longan varieties can be divided into either a subtropical longan group or a tropical longan group [21]. Most cultivated longan varieties, such as “SX”, one of the main varieties in China, belong to the subtropical longan group. Similar to other perennial fruit trees, these kinds of longan trees require an appropriate period of low temperatures for bud break or good floral induction [22]. According to a previous study, a duration of 4–6 weeks of low temperatures of approximately 15–20 °C is necessary for the floral induction or bud break of these kinds of longan varieties [23]. In contrast, longan varieties such as SJ, which belong to the tropical longan group, exhibit the “perpetual flowering” (PF) habit, and a period of low temperatures is not a necessary condition for bud break or floral induction in these kinds of longan trees. Thus, “SJ” is a good material for studying the genetic and physiological bases of bud break.

Although several reports have studied floral induction in “SJ” using RNA-seq analysis [24, 25], the molecular mechanism of bud break and floral induction of “SJ” remains unknown. In addition, according to previous studies, the correlation between transcript abundance and protein concentration is poor due to translation regulation [26]. Proteins are the direct performers of vital movement [27]. Therefore, the mechanism of biological processes cannot be analyzed using transcriptome sequencing alone. Proteomes need to be investigated to provide a better understanding of the molecular mechanism of bud dormancy release and floral induction in SJ. In the present study, a comparative proteomic analysis was performed of “SX” and “SJ” during three floral induction stages using iTRAQ technology, which has successfully been used in *Arabidopsis*, citrus, and *Camellia oleifera* [28, 29]. Our goal was to elucidate the molecular mechanism of the floral induction of SJ, especially bud break, at the proteome level and to identify the important proteins involved in bud break.

## Materials and methods

### Plant materials

“SX” and “SJ” longan trees were both cultivated in the same orchard located in Mazhang district (110°16′ E, 21°10′ N), Zhanjiang, Guangdong Province, P. R. China (the identification was undertaken by Pro. Wang [30]). Three developmental flower bud samples were obtained during different periods from November 2016 to January 2017: the dormant apical bud period (T1) (November 20, 2016), the floral primordia differentiation period (red bud) (T2) (December 24, 2016), and the floral organ formation period (T3) (January 1, 2017) (Fig. S1). For each phase, uniform buds were pooled and divided into quarters for transcriptome sequencing, proteome profiling, qRT‒PCR verification, and sugar assays. The samples were frozen immediately in liquid nitrogen and stored at ‒80 °C.

### Measurements of soluble sugars and starch

The contents of soluble sugars in different flower bud samples were determined using high-performance liquid chromatography (HPLC) (LC-20 A, Shimadzu Corp., Kyoto, Japan) following the description by Shi et al. [31]. In short, a 2 g flesh sample was mixed and homogenized with 10 mL distilled water and incubated at 37 °C for 30 min. After centrifugation at 5000×g for 10 min, the supernatant was evaporated to dryness at 75 °C in a water bath. The residue was dissolved in 5 mL distilled water and filtered before analysis. An analysis of soluble sugars was carried out using an amino column (250 mm × 4.6 mm; Kromasil, Bohus, Sweden) with a flow rate of 1.0 mL·min^− 1^ at 35 °C. For the mobile phase, acetonitrile and twice-distilled water (70:30 v/v) were used along with a refractive index detector. The starch contents of the different flower bud samples were determined enzymatically as glucose equivalents following the method proposed by Chow et al. [32].

### Protein extraction, iTRAQ labeling, and proteomics analysis

The total proteins of the flower bud samples of “SX” and “SJ” were extracted following the method of phenol extraction described by Chen et al. [33] with slight modification. In brief, 1 g of each bud sample was finely ground to a powder with nitrogen and polyvinylpyrrolidone (PVPP) and suspended in a two-phase system consisting of fresh extraction buffer and chilled phenol buffered with Tris (hydroxymethyl) aminomethane hydrochloride (Tris-HCl), pH 7.8. Then, the homogenate was centrifuged at 7100 × g for 10 min at 4 °C. The phenol-based upper phase was transferred to a new conical tube, mixed with five volumes of precooled methanolic 0.1 M ammonium acetate and incubated at − 20 °C overnight. The precipitates were collected and washed with ice-cold methanol and acetone to remove interfering compounds. Next, each pellet was solubilized in sodium dodecyl sulfate (SDS) lysis buffer at room temperature for approximately 3 h. A Bovine Serum Albumin Protein Assay Kit (Thermo Fisher, USA) was used to quantify the final protein solution. The quality and integrity of the protein were evaluated using SDS‒PAGE. iTRAQ labeling and analysis were implemented by the GENE DENOVO Company, Guangzhou, China. Three independent biological replicates were performed. iTRAQ reagents (iTRAQ® Reagent-8PLEX Multiplex Kit, Sigma) were used for iTRAQ labeling. A shotgun proteomics analysis was performed using the EASY-nLCTM 1200 UHPLC system (Thermo Fisher, Shanghai, China) and an Orbitrap Q Exactive HF-X mass spectrometer (Thermo Fisher, Shanghai, China) operating in the data-dependent acquisition (DDA) mode. Proteins were identified using the sequenced longan genome [34]. The Mann–Whitney test was used to perform a statistical analysis of the protein quantification results, and significant ratios were defined as p value ≤ 0.05, fold change ≥ 1.2 (upregulation of protein expression), and fold change ≤ 0.83 (downregulation of protein expression), which were used to screen differentially abundant proteins (DAPs). We searched against the GO and KEGG databases to classify and identify differentially expressed proteins. Significant pathway enrichment was examined with the hypergeometric test, and significance was set at p < 0.05.

### Integrated transcriptome and proteome analysis

RNA sequencing data were obtained in our previous study [30]. To investigate the concordance between the transcript and protein levels, Pearson correlation tests were conducted based on the log_2_-fold change in DEGs and DAPs during different flower induction stages in both accessions.

### Gene expression validation

The qRT‒PCR materials and methods are shown in our previous study [30]. The qRT‒PCR primers are shown in Table S1.

**Fig. 1:**
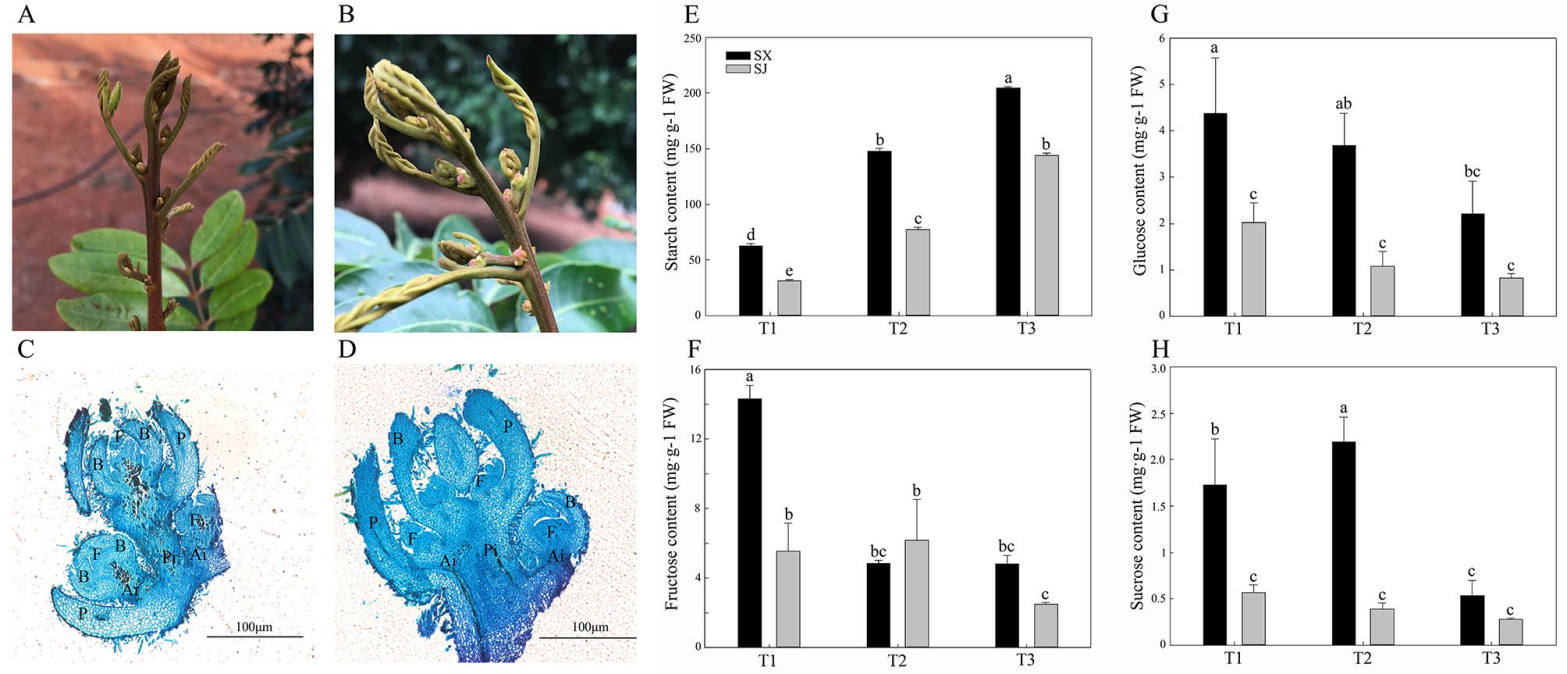
Microphotograph and sugar contents of flower buds of SJ and SX longan species. (A) Flowering traits of “SJ”. (B) Flowering traits of “SX”. (C) Microphotograph of the T_2_-stage flower bud of SJ. (D) Microphotograph of the T_2_-stage flower bud of “SX”. (E) Starch content. (F) Fructose content. (G) Glucose content. (H) Sucrose content. Ai = axillary inflorescence; B = bract; F = floral primordium; P = prophyll; Pi = primary inflorescence. Values are the means of three replicates ± SE

## Results

### Flowering phenotype and sugar content in “SX” and “SJ” longan buds during flower induction

“SX” is a typical “seasonal flowering” (SF) longan cultivar, and it needs an appropriate period of low temperatures to accumulate energy and nutrients for flower induction. Because of these traits, its inflorescence and postdormant bud develop better and more robustly (Fig. 1 A). Different from “SX”, an appropriate environmental factor is not necessary for bud break or floral induction of “SJ”. Therefore, “SJ” can flower throughout the whole year, even in adverse environmental conditions, such as low temperatures in summer and high temperatures in winter. The dormant buds, flowers, and fruits of SJ can appear on the same branch at the same time. However, because of its PF habit, the flowers and fruits of “SJ”were always smaller and weaker than those of “SX” (Fig. 1B). The anatomical analysis results showed that, when comparing the buds of these two typical longan cultivars, “SX” was composed of a primary inflorescence that was stronger than that of “SJ”, and its axillary inflorescence was composed of a floral primordium, bract, and prophyll that were better developed than those of “SJ”, which means that “SX” reserves more energy for flowering and fruiting (Fig. 1 C and 1D). To better understand the physiological basis of the bud dormancy release of SJ, the contents of four kinds of sugars, namely, starch, fructose, glucose, and sucrose, were measured. Consistent with the phenotypic and anatomical analyses, most of the sugars, except for fructose, were higher in “SX” than in “SJ” during flower induction (Fig. 1E-H). Among these sugars, the contents of starch, glucose, and sucrose in T2 of “SX” were 1.9, 3.4, and 5.6 times higher than those in T2 of “SJ”, respectively. Additionally, the starch content increased in “SX” and “SJ” during flower induction. The fructose content decreased in “SX” during the T1 to T2 transition. The glucose and sucrose contents decreased in “SX” and “SJ” during flower induction.

### General characterization of proteomic data

**Fig. 2:**
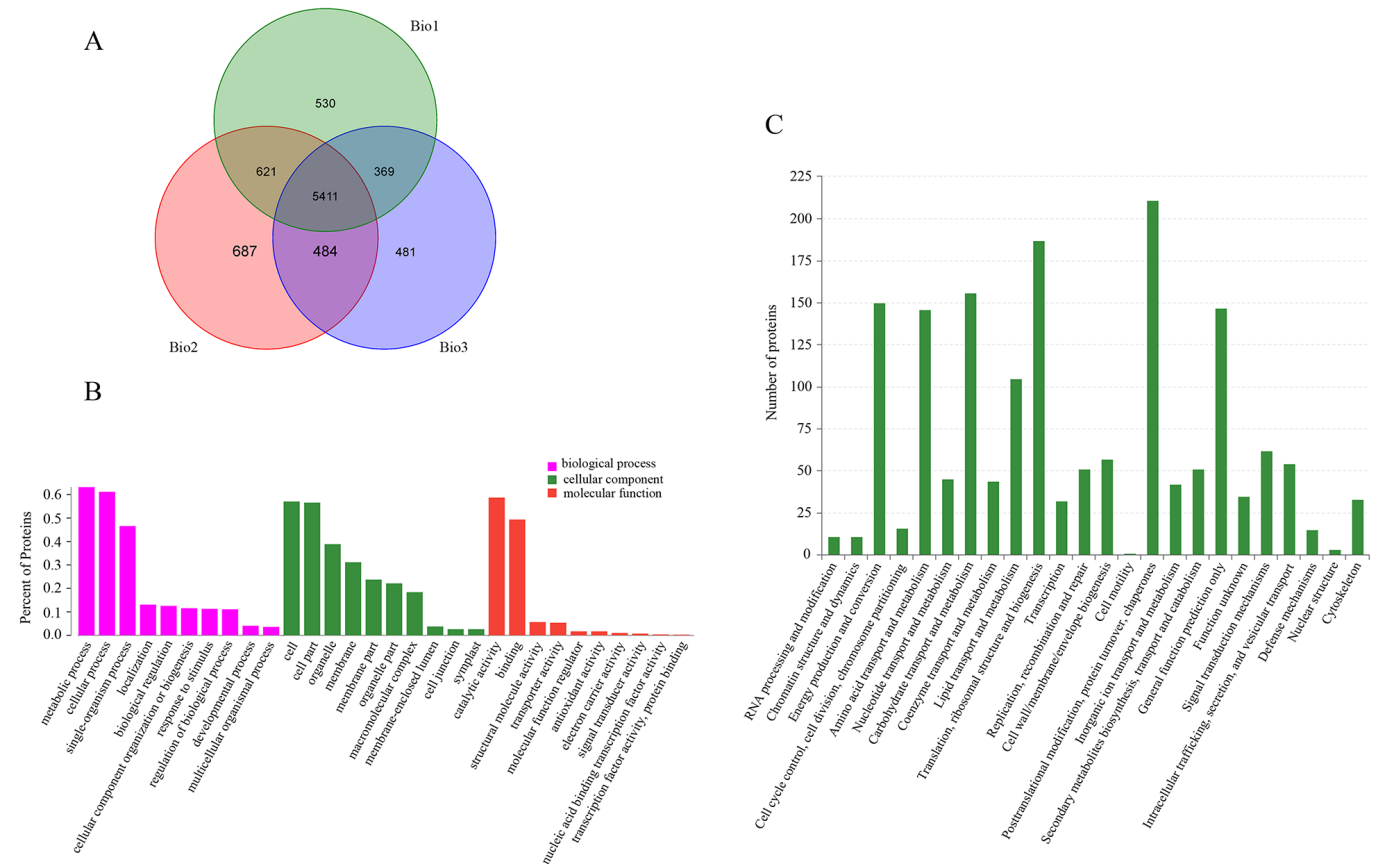
Venn diagram, GO enrichment analysis, and COG analysis of proteins in three biological experiments, including 18 flower bud samples of SJ and SX longan species. (A) Venn diagram of three biological experiments. Bio1, Bio2, Bio3 represent the three biological experiments; (B) GO enrichment analysis of 3180 unique protein species that matched at least two unique peptides; (C) COG analysis of 3180 unique protein species that matched at least two unique peptides

To further study the reason for the different flowering traits of “SX” and “SJ”, a comparative proteome survey was performed on “SX” and “SJ” using the iTRAQ technique. Raw data were deposited into the ProteomeXchange Database (accession number: PXD006710). A total of 419,206, 401,957, and 392,040 spectra were generated in the three biological experiments. A total of 6139, 6407, and 5935 proteins were matched to the longan protein database (Fig. 2a and Supplementary Table S2). After merging these data, a total of 5411 nonredundant proteins were identified in the three independent biological replicates (Fig. 2a). Among these 5411 proteins, 3180 unique protein species that matched at least two unique peptides were selected for further analysis (Table S3). Additionally, the distributions of the peptide length, number, mass, and sequence coverage of the proteins in the three replicates were assessed (Figure S2-S4).

According to Gene Ontology (GO) enrichment analysis, 2731 of 3180 proteins were classified into three groups (Fig. 2b). The main cellular components were cells (57.05%), cell parts (56.54%), membranes (31.12%), organelles (38.85%), and other components. The molecular functions of the proteins were mainly focused on binding (49.36%), catalytic activity (58.73%), and other functions. The biological processes were classified into metabolic processes (63.16%), cellular processes (61.19%), single-organism processes (46.58%), and other processes (Table S4). Meanwhile, 1581 of all 3180 proteins could be assigned to 24 categories using the Cluster of Orthologous Groups of Proteins (COG) database (Fig. 2c). Posttranslational modification, protein turnover, and chaperones (13.35%, n = 211) made up the largest group, followed by translation, ribosomal structure, and biogenesis (11.83%, n = 187); carbohydrate transport and metabolism (9.87%, n = 156); energy production and conversion (9.49%, n = 1150); and others (Table S5).

### Identification and KEGG pathway enrichment analysis of DAPs

**Fig. 3:**
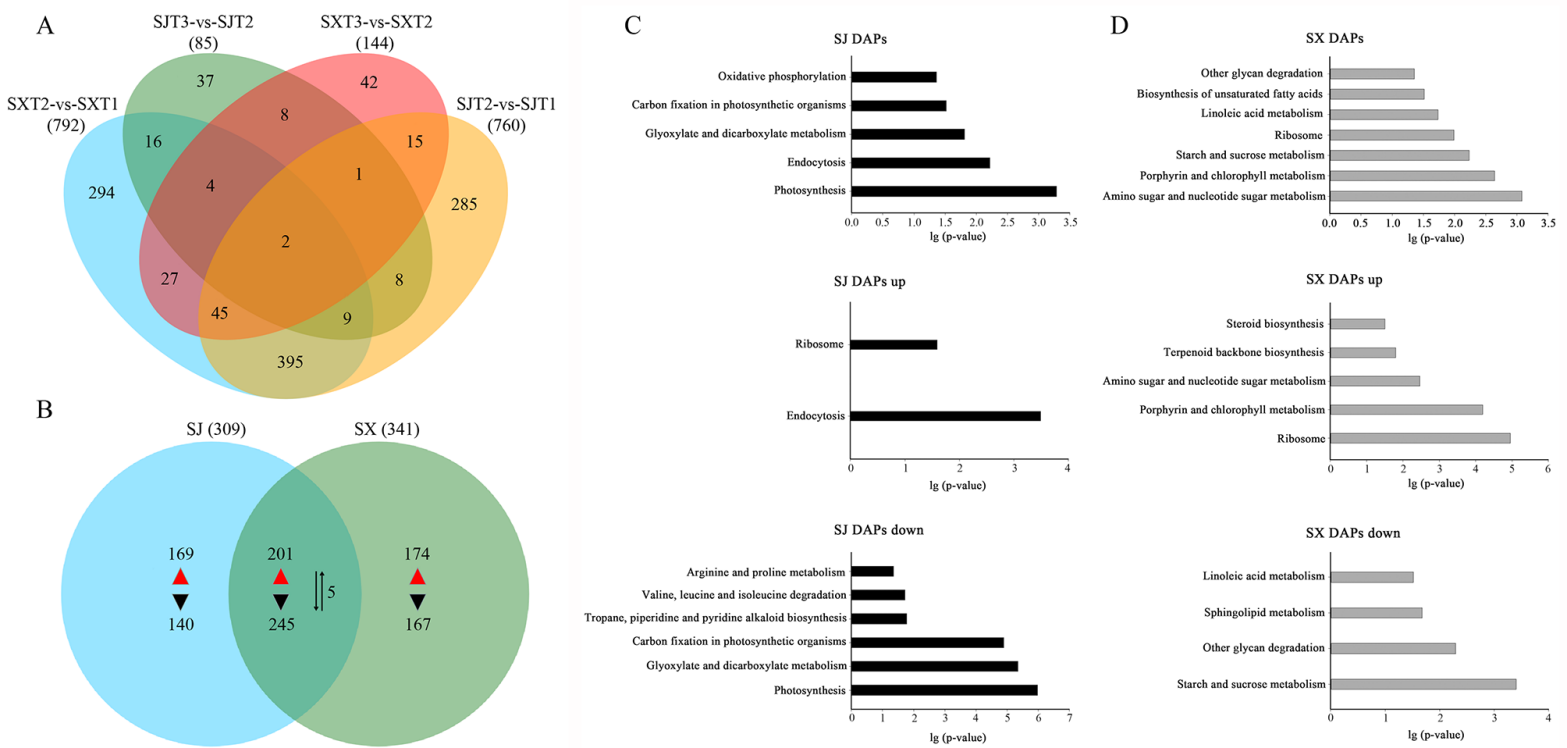
DAP identification and KEGG pathway enrichment analysis in “SJ” and “SX” during flower induction. (A) Venn diagram showing the number of DAPs in “SJ” and “SX” during the flower induction process. (B) Venn diagram showing the number of up- and downregulated DAPs in “SJ” and “SX” during the bud dormancy release process. (C) The specifically enriched KEGG pathways, including up- or down-regulated, of “SJ”. (D) The specifically enriched KEGG pathways, including up- or down-regulated, of “SX”

Proteins that underwent a 1.2-fold or 0.83-fold change in abundance with P value < 0.05 between two time points in a particular species (T1–T2 and T2–T3) were identified as DAPs. As shown in Fig. 3 A, the distributions of the changes were biased toward the early floral induction stage in both accessions (FDR < 0.05): 755 and 787 DEPs were identified in SJT1-vs.-SJT2 and SXT1-vs.-SXT2, respectively. However, only 85 and 144 DEPs were found in “SJ” and “SX”, respectively, during the T2 to T3 transition stage (Fig. 3 A). This result indicated that, at the protein level, regulation in the early floral induction stage (bud break) (T1-T2) was much more complex than that in the flower formation period (T2-T3) in the two longan varieties. Considering that the research goal of this study was to analyze the protein basis of longan bud break, 1101 DAPs identified in SJT1-vs.-SJT2 and SXT1-vs.-SXT2 were selected for further analysis (Fig. 3B and TableS6).

Among the 1101 DAPs, 544 were upregulated DAPs (more abundant in the stage of the emergence of floral primordia than in the dormant stage), 552 were downregulated DAPs, and 5 DAPs displayed contrasting patterns in the two longan species. The number of DAPs and the overlaps between the two longan species are summarized in Table 1. We observed that 446 DAPs, namely, 201 upregulated and 245 downregulated proteins, had similar patterns in the two longan species. “SX” had 341 unique DAPs, namely, 174 upregulated and 167 downregulated DAPs, and “SJ” had 309 unique DAPs, namely, 169 upregulated and 140 downregulated DAPs (Fig. 3B and Table S7). Our findings indicate that these proteins may play critical roles in floral induction and the formation of PF traits in longan. To further analyze the pathways specifically enriched in the different longan species, a KEGG analysis was performed. For these 309 “SJ” unique DAPs, five pathways were enriched, namely, photosynthesis, endocytosis, glyoxylate and dicarboxylate metabolism, carbon fixation in photosynthetic organisms, and oxidative phosphorylation. Among these pathways, photosynthesis, glyoxylate and dicarboxylate metabolism, and carbon fixation in photosynthetic organisms, which are related to energy metabolism, were all enriched in the downregulated “SJ” DAPs (Fig. 3 C and Table S8). Seven pathways (amino sugar and nucleotide sugar metabolism, porphyrin and chlorophyll metabolism, starch and sucrose metabolism, ribosome, linoleic acid metabolism, biosynthesis of unsaturated fatty acids, and other glycan degradation) were enriched in “SX”. Among these pathways, starch and sucrose metabolism, linoleic acid metabolism, and other glycan degradation were enriched in the downregulated “SX” DAPs, while amino sugar and nucleotide sugar metabolism and porphyrin and chlorophyll metabolism were enriched in the upregulated “SX” DAPs (Fig. 3D and Table S9).

**Table 1:** The number of DAPs in SJ and SX during bud dormancy release

	“SJ”	“SX”	overlapped	
			**similar pattern**	**contrasting pattern**
Upregulated	370	375	201	5 (SX)
Downregulated	385	412	245	5 (SJ)
Unique	309	341	–	-
Total	755	787	-	451

### Integrative analysis of the proteome and transcriptome during bud break

**Fig. 4:**
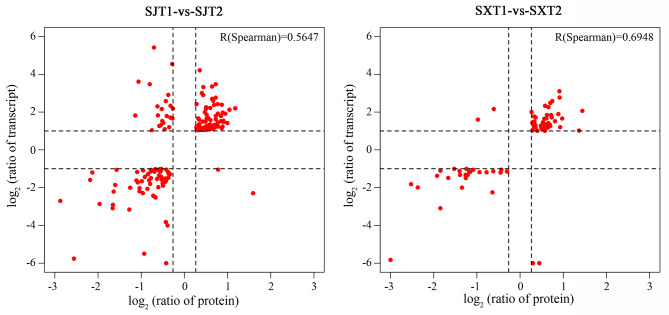
Correlation between the proteome and transcriptome in “SJ” and “SX” during bud break

In the present study, a transcriptomic analysis of the same samples used in iTRAQ was performed using the RNA-seq method [30], allowing for a comparison of transcript and protein expression during longan bud break. The results showed that 164 and 77 DAPs were successfully matched with DEGs in the SJT1-vs.-SJT2 and SXT1-vs.-SXT2 pairs, respectively (Fig. 4 and Tables S10 and S11). A Pearson correlation test showed that the corresponding Spearman correlation coefficients for the proteome and transcriptome were 0.5647 and 0.6948, respectively. Among these DEGs/DEPs, 76 in SJT1-vs.-SJT2 and 46 in SXT1-vs.-SXT2 were upregulated at both the transcript and protein levels; 62 in SJT1-vs.-SJT2 and 26 in SXT1-vs.-SXT2 were downregulated at both the transcript and protein levels; 2 and 2 in SJT1-vs.-SJT2 and SXT1-vs.-SXT2 were upregulated and downregulated, respectively; and 24 and 3 in SJT1-vs.-SJT2 and SXT1-vs.-SXT2 were downregulated and upregulated, respectively. This result suggested that the regulatory mechanisms are different at the mRNA and protein levels and that massive posttranscriptional regulation may exist during bud break in SJ and SX.

### DAPs involved in bud break of longan

**Fig. 5:**
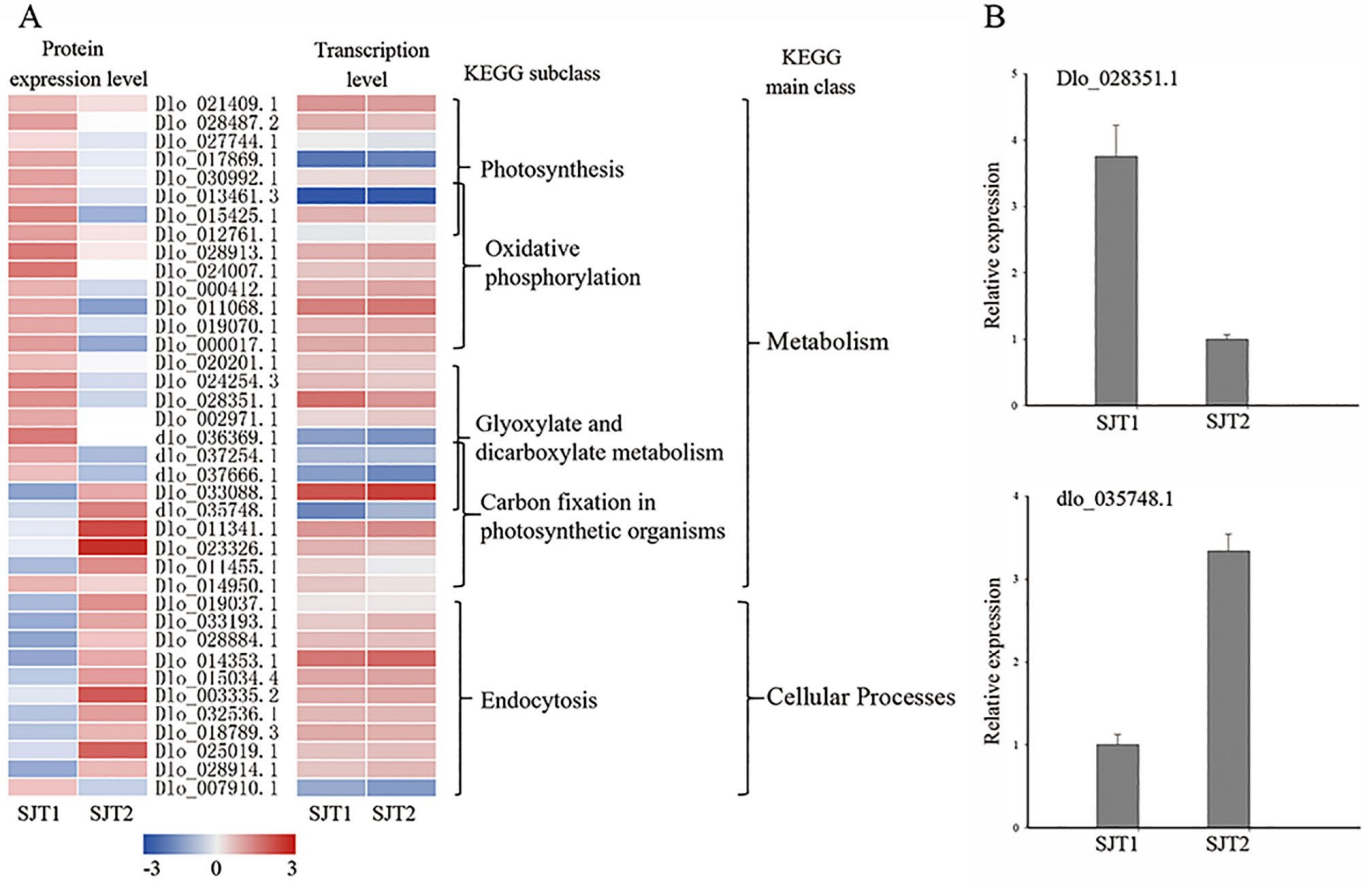
The unique DAPs involved in bud dormancy release regulation of “SJ”. (A) Heatmap of the unique DAPs involved in bud dormancy release regulation of SJ. Data for gene and protein levels were normalized by the Z score. Red and blue indicate up- and downregulated DAPs, respectively. (B) Validation of the expression of the unique DAPs involved in bud dormancy release regulation of “SJ” by qRT–PCR analysis. Error bars indicate the standard deviation of three biological replicates

To better understand the DAPs involved in the bud break of longan, the unique DAPs belonging to the pathways specifically enriched in the different longan species were analyzed based on our iTRAQ data and RNA-seq data. In total, 38 DAPs that were enriched in “SJ” belonged to five pathways, namely, photosynthesis (8), endocytosis (11), glyoxylate and dicarboxylate metabolism (9), carbon fixation in photosynthetic organisms (9), and oxidative phosphorylation (10) (Fig. 5 A and Table S8). Among these pathways, all of the proteins involved in photosynthesis, glyoxylate and dicarboxylate metabolism, and oxidative phosphorylation were significantly downregulated during bud break. Among these 18 downregulated DAPs, only one protein (catalase isozyme 1, Dlo_028351.1) was downregulated at both the transcript and protein levels. In the carbon fixation in photosynthetic organisms pathway, three ribulose bisphosphate carboxylase large-chain (RuBisCO large subunit) proteins and one phosphoribulokinase, chloroplastic (PRK) protein were significantly downregulated during the bud break process; and ribulose bisphosphate carboxylase small-chain, ribulose bisphosphate carboxylase large-chain, glyceraldehyde-3-phosphate dehydrogenase A, transketolase, chloroplastic (TK), and sedoheptulose-1,7-bisphosphatase (SBPase) were significantly upregulated during the bud break process. Among these nine DAPs, only one (ribulose bisphosphate carboxylase large chain, dlo_035748.1) was upregulated at both the expression transcript and protein levels. Most DAPs in the endocytosis pathway were upregulated during bud break at the protein level. Only one protein (phospholipase D alpha 1, PLD 1) was downregulated. None of the proteins in this pathway showed significant expression at the transcript level. In addition, the DAPs downregulated or upregulated at both the transcript and protein levels were verified by qRT‒PCR (Fig. 5B).

**Fig. 6:**
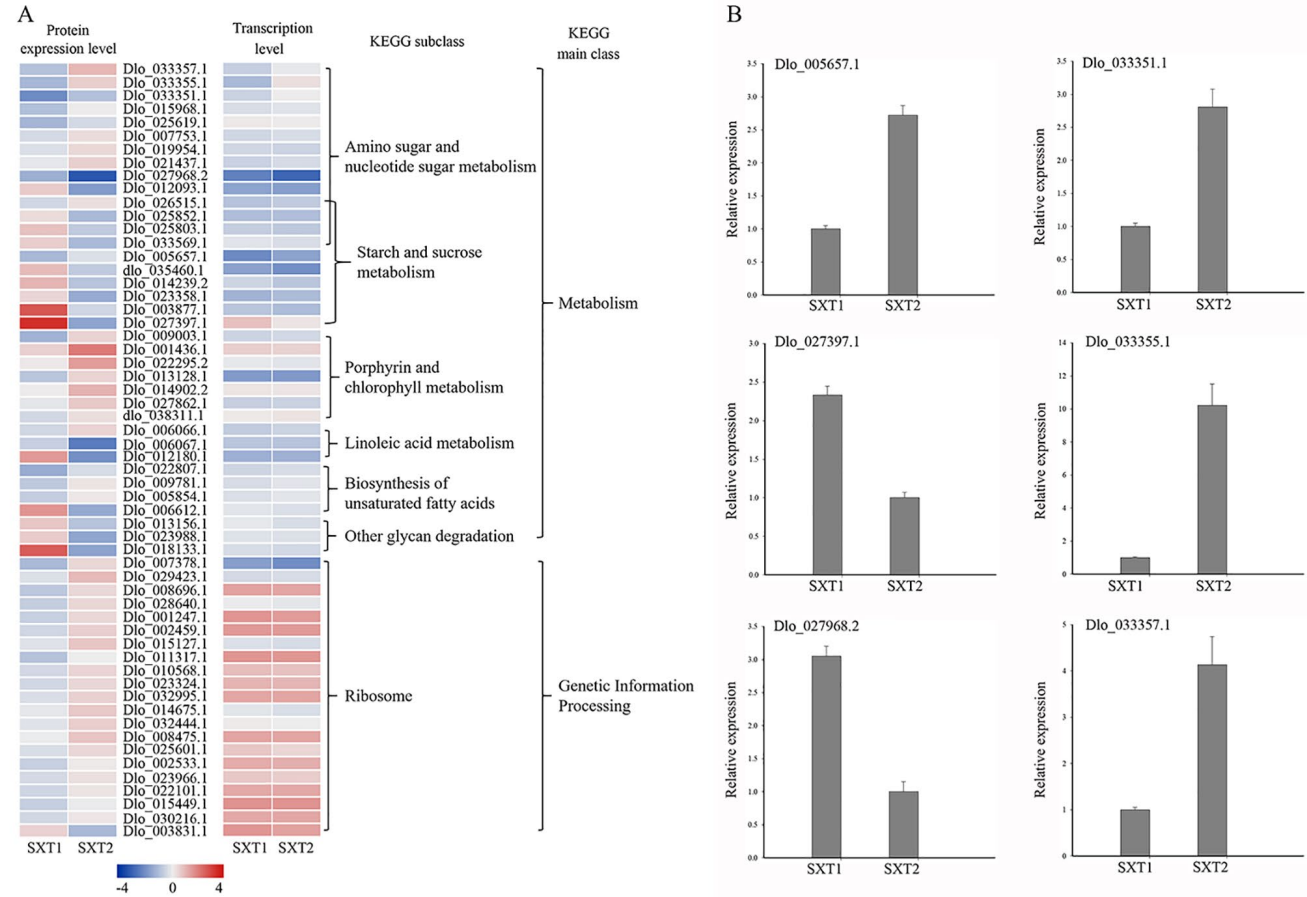
The unique DAPs involved in bud break regulation of ‘SX’. (A) Heatmap of the unique DAPs involved in bud dormancy release regulation of “SX”. Data for gene and protein levels were normalized by the Z score. Red and blue indicate up- and downregulated DAPs, respectively. (B) Validation of the expression of the unique DAPs involved in bud dormancy release regulation of “SX” by qRT–PCR analysis. Error bars indicate the standard deviation of three biological replicates

During the bud dormancy release of “SX”, we found 58 DAPs enriched in seven pathways, namely, amino sugar and nucleotide sugar metabolism (14), porphyrin and chlorophyll metabolism (7), starch and sucrose metabolism (10), ribosome (21), linoleic acid metabolism (3), biosynthesis of unsaturated fatty acids (4), and other glycan degradation (3) (Fig. 6 and Table S9). Among these pathways, all of the DAPs in porphyrin and chlorophyll metabolism were upregulated during bud break, and all of the DAPs in other glycan degradation were downregulated during bud break. Our study identified eight amino sugar and nucleotide sugar metabolism-related proteins, namely, endochitinase PR4 (Dlo_033357.1), chitinase 4 (Dlo_033355.1), chitinase 5 (Dlo_033351.1), GDP-L-fucose synthase 1 (Dlo_015968.1), phosphomannomutase (GmPMM) (Dlo_025619.1), UDP-arabinopyranose mutase 5 (Dlo_007753.1), UDP-glucuronic acid decarboxylase 4 (UGD) (Dlo_019954.1), and GDP-mannose 4,6 dehydratase 1 (GMD 1) (Dlo_021437.1), that were upregulated during bud break, and two proteins, namely, chitinase 4 and probable beta-D-xylosidase 5, were downregulated. In the starch and sucrose metabolism pathway, most of the proteins, including two glucose-1-phosphate adenylyltransferase proteins, one glucose-6-phosphate isomerase protein, two 4-alpha-glucanotransferase proteins, one isoamylase 3 (ISA3) protein, one beta-fructofuranosidase protein, and one granule-bound starch synthase 1 (GBSS-I) protein, were downregulated, and only two proteins, namely, sucrose synthase 6 (SUS6) and fructokinase-5, were upregulated. For the biosynthesis of unsaturated fatty acid-related proteins, three proteins, namely, two enoyl-CoA reductase (ECR) proteins and one acyl-coenzyme An oxidase 3 (AOX3) protein, were upregulated, and one short-chain-type dehydrogenase was downregulated. In the ribosome pathway, most of the proteins were upregulated, and only one protein (60 S acidic ribosomal protein P0) was downregulated. In addition, among these 58 DAPs, only four amino sugar and nucleotide sugar metabolism-related proteins (one endochitinase PR4 protein, Dlo_033357.1; one chitinase 5 protein, Dlo_033351.1; and two chitinase 4 proteins, Dlo_033355.1 and Dlo_027968.2) and two starch and sucrose metabolism-related proteins (SUS6, Dlo_005657.1 and GBSS-I, Dlo_027397.1) were up- and downregulated at the transcript and protein levels. Interestingly, all six DAPs belonged to the group of carbohydrate metabolism proteins. In addition, the DAPs downregulated or upregulated at both the transcript and protein levels were verified by qRT‒PCR (Fig. 6B).

## Discussion

To date, the genetic control of bud break has been well studied in model plants [35]. However, the multiyear delay in the onset of flowering and the long juvenile phase hamper studies of bud break in perennials. Our previous study elucidated the regulatory mechanisms involved in the bud break of longan at the transcription level [30]. However, many studies have shown that transcript abundance only partially predicts protein abundance and that a series of regulatory processes involved in translation, localization, modification, and protein degradation play a substantial role in controlling protein expression [36]. Therefore, expression changes detected at the mRNA level may or may not result in variable protein abundances as controlled by protein turnover, while at the same time, expression changes at the protein level may or may not also be observed at the mRNA level [37]. For example, in the research conducted by Xanthopoulou et al. [37], only 29 tissue-specific protein-coding genes (8.8%) were validated using their integrated proteogenomic approach. Ye et al. [29] found that more than 98% of DAPs were covered by their transcriptomic results. However, the concordance between the expression levels of DEGs and DAPs was very poor. Therefore, an integrative analysis of transcriptomes and proteomes can serve as an effective tool for obtaining information concerning specific biological reactions and as a powerful technique for identifying the proteins responsible for regulating the metabolic pathways involved in bud break. In our research, a total of 3180 unique proteins were identified. Consistent with previous studies [38], GO and COG enrichment analyses showed that most of the proteins could be classified into metabolic processes (63.16%) and cellular processes (61.19%). Posttranslational modification, protein turnover, and chaperones (13.35%, n = 211) formed the largest group, followed by translation, ribosomal structure, and biogenesis (11.83%, n = 187); carbohydrate transport and metabolism (9.87%, n = 156); and energy production and conversion (9.49%, n = 1150), which suggests that energy metabolism-related proteins and posttranslational modification may be crucial during the flower development of longan. The largest number of DAPs was detected in the comparison of SJT1-vs.-SJT2 and SXT1-vs.-SXT2, implying that greater changes in biological processes may appear in bud break phases. In addition, consistent with previous studies [29], the concordance between the expression levels of DEGs and DAPs was very poor in our study. These results indicated that abundant posttranslational modification existed during bud break phases, and they prove the importance of analyzing specific biological reactions at the transcript and protein levels.

Different from “SJ”, “SX” belongs to typical SF longan cultivars, and it needs winter chilling (an appropriate period of low temperatures) to meet its requirements to induce flowering [39]. This flowering trait means that “SX” has a normal growth cycle and more dormancy time for the development of flower buds. Consistent with the flowering trait, our results showed that the buds of “SX” were composed of primary inflorescences and axillary inflorescences that are stronger than those of “SJ”, which indicated that the flower bud development of “SX” was better than that of “SJ”, and its preparation was also better for subsequent flowering. Additionally, our results showed that “SX” contains higher contents of starch, glucose, and sucrose in T1 and T2, implying that “SX” has to accumulate more assimilates and energy since a whole winter’s dormancy for bud break and flowering. It has previously been found that available carbohydrates and starch accumulated before flower initiation and leaf flushing in both lychee and longan [2]. In addition, we found that the starch content was increased in “SX” during flower induction. The fructose content was decreased in “SX” during the T1 to T2 transition. The glucose and sucrose contents were decreased in “SX” flowers during induction. Similar to our research, previous studies have shown that sugars regulate growth and flowering transition in grape [40], citrus [41], and apple [42].

Consistent with our anatomical analysis and physiological analysis results, we found that most DAPs in “SX” during the bud break process were enriched in assimilates and energy metabolism-related proteins. Numerous experiments have indicated that energy metabolism-related proteins play important roles in flower bud development [7, 13, 14]. In addition, the key DAPs, whose expression trend was consistent at the transcriptional level and protein level, all belonged to a group of carbohydrate metabolism-related proteins. Among these DAPs, chitinases, which are usually induced in the pathogen response [43], are upregulated at the transcript and protein levels in “SX” during the bud dormancy release phases. Similarly, several studies have found that chitinases also increase during flower development [44, 45]. Starch metabolism and biosynthetic processes are involved in flower induction [46]. A previous study found that the starch content in buds increased during the flower induction process of apple and that the expression levels of sucrose synthase 4 (SS4) and granule-bound starch synthase 1 (GBSSI), which are associated with the starch biosynthesis process in buds, displayed similar changes [42]. Similar to this study, sucrose synthase 6 (SS6) was upregulated at the transcript and protein levels in “SX” during the bud dormancy release phases. However, GBSSI was downregulated. These results show that sugars (as energy substances) and their synthesis and metabolism-related proteins are important factors during bud break in “SX”.

Compared to “SX”, the bud break or floral induction of “SJ”, a typical SF longan cultivar, does not need an appropriate environmental factor. Therefore, “SJ” can flower and fruit throughout the year. Due to this trait, “SJ” usually has smaller fruit and lower yields [47]. Although many studies have investigated flower induction in SJ at the physiological and transcriptional levels [24], the regulatory mechanism of SJ has still not been clarified. Consistent with previous observations of fruit size and yield, we found that the primary inflorescence, axillary inflorescence, floral primordium, bract, and prophyll of “SJ” were weaker than those of “SX”. In addition, almost all of the tested sugar contents in SJ were lower than those in SX during the bud break phases. These results indicated that although “SJ” can overcome the biennial bearing problem, its bud development was worse than that of “SX”, and it only requires a minimum amount of energy to maintain flowering and fruiting. Our proteomic analysis showed that the DAPs involved in bud break of SJ were enriched in five pathways, and four of these pathways belonged to energy metabolism-related proteins. In addition, all of the DAPs involved in photosynthesis, glyoxylate and dicarboxylate metabolism, and oxidative phosphorylation and almost all of the DAPs involved in carbon fixation in photosynthetic organisms were downregulated during the bud break phases of “SJ”. Interestingly, photosynthesis-related proteins were downregulated during longan flowering reversion [4]. Similar to sucrose, light can act as both the source and signal of energy for bud growth, and an increased light intensity can accelerate budburst in a range of species [48]. In many plants, photoperiods can interact with temperature to control flowering. For example, the start of spring, which has warmer temperatures and longer photoperiods, brings hop out of dormancy [49]. Furthermore, Bauerle (2019) found that photoperiods are the sole environmental trigger for the flower initiation of hop, whereas low-temperature chilling and dormancy are not triggers for the flower initiation of hop [49]. Recently, a study found that a longer photoperiod can offset insufficient chilling in some subtropical trees [10]. Common longan varieties, such as “SX”, are long-day plants, and they require a higher Pfr/Pr ratio to flower [50]. After dormancy and chilling, the warm temperature and longer photoperiods trigger bud break. However, the high temperatures and high-intensity light in summer are adverse environmental conditions for flowering, especially for bud break. In “SJ”, photosynthesis-related and oxidative phosphorylation-related DAPs, such as photosystem I reaction center protein, ATP synthase, and NADH dehydrogenase, which are important components of photosynthetic phosphorylation, were downregulated during the bud break phases of “SJ”. These results indicate that, although light is an important signal for triggering bud break in common longan cultivars (such as “SX”), “SJ” may not be sensitive to light intensity changes in the external environment.

Two proteins were identified as specifically being upregulated (ribulose bisphosphate carboxylase large chain, dlo_035748.1) or downregulated (catalase isozyme 1, Dlo_028351.1) at the transcript and protein levels in “SJ” during the bud dormancy release phases. The first protein is catalase isozyme 1. Catalase (CAT) is an important enzyme in the redox cycle and is a heme-containing compound [51]. Previous studies have proven that catalase isozymes can be induced by various environmental stressors in many plants and that some detoxification pathways are upregulated during dormancy release, including catalase (CAT) [52]. The second protein is the ribulose bisphosphate carboxylase (RuBisCO) large-chain protein. RuBisCO is a key enzyme in the Calvin cycle of photosynthetic carbon assimilation in plants. A previous study showed that RuBisCo was downregulated in the flowering reversion of longan [50]. Similar to these studies, our results showed that CAT and RuBisCo may be important factors for the bud break of SJ.

## Conclusion

Our results showed that the key DAPs and enriched pathways in these two longan varieties during the bud break process were quite different based on physiological, anatomical, transcriptome, and proteome analyses. For “SX”, assimilates and energy-metabolism-related pathwayswere important during the bud break process, and the key proteins are starch- and sucrose-metabolism-related proteins (SUS6 and GBSS-I). Different from “SX”, light, rather than a high sugar content, dormancy, or chilling duration, might act as the key signal for triggering the bud break of “SJ”. Most DAPs were enriched in photosynthesis-related pathways, and the key proteins were catalase isozyme 1 and RuBisCO. Taken together, our findings provide a better understanding of the complex regulatory mechanism underlying bud break in longan.

## Electronic supplementary material

Below is the link to the electronic supplementary material.

Supplementary Material 1

Supplementary Material 2

Supplementary Material 3

Supplementary Material 4

Supplementary Material 5

Supplementary Material 6

Supplementary Material 7

Supplementary Material 8

Supplementary Material 9

Supplementary Material 10

Supplementary Material 11

Supplementary Material 12

## Data Availability

The datasets generated and/or analysed during the current study are available in the NCBI SRA database (http://www.ncbi.nlm.nih.gov/sra) with the accession number: SRS2241241–SRS2241258. Raw data of iTRAQ were deposited on the ProteomeXchange Database (accession number: PXD006710).

## Declarations

### Statement

Our experimental research on “SX” and “SJ” longan was in accordance with the relevant national/institutional guidelines.
