# Supplementary material for: A comparative proteomic analysis provides insight into the molecular mechanism of bud break in longan
Source: BMC Plant Biol. 2022 Oct 12;22:486. doi: 10.1186/s12870-022-03868-3 (PMC9558362; doi:10.1186/s12870-022-03868-3)
Supplement: Supplementary file 1 — Supplementary Material 1 [file 12870_2022_3868_MOESM1_ESM.docx]

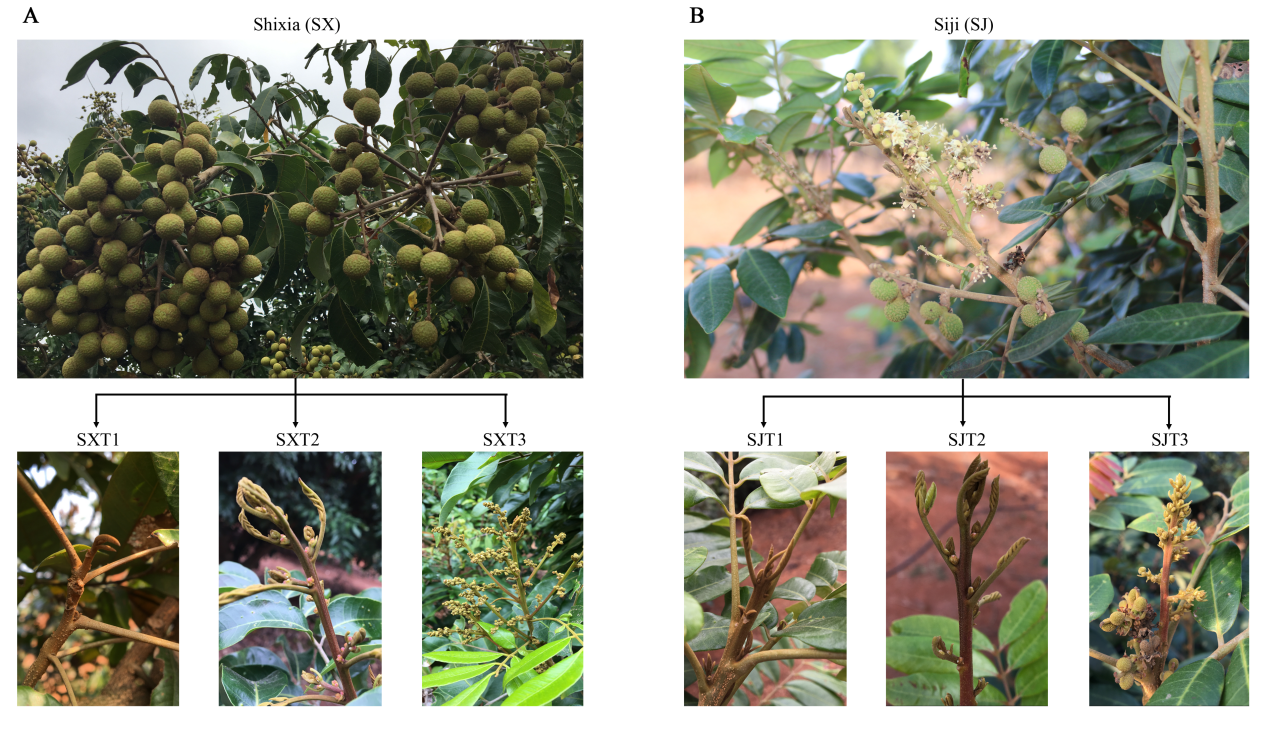


Fig S1 Different flowering phenotypes of “SX” and “SJ” longan trees.


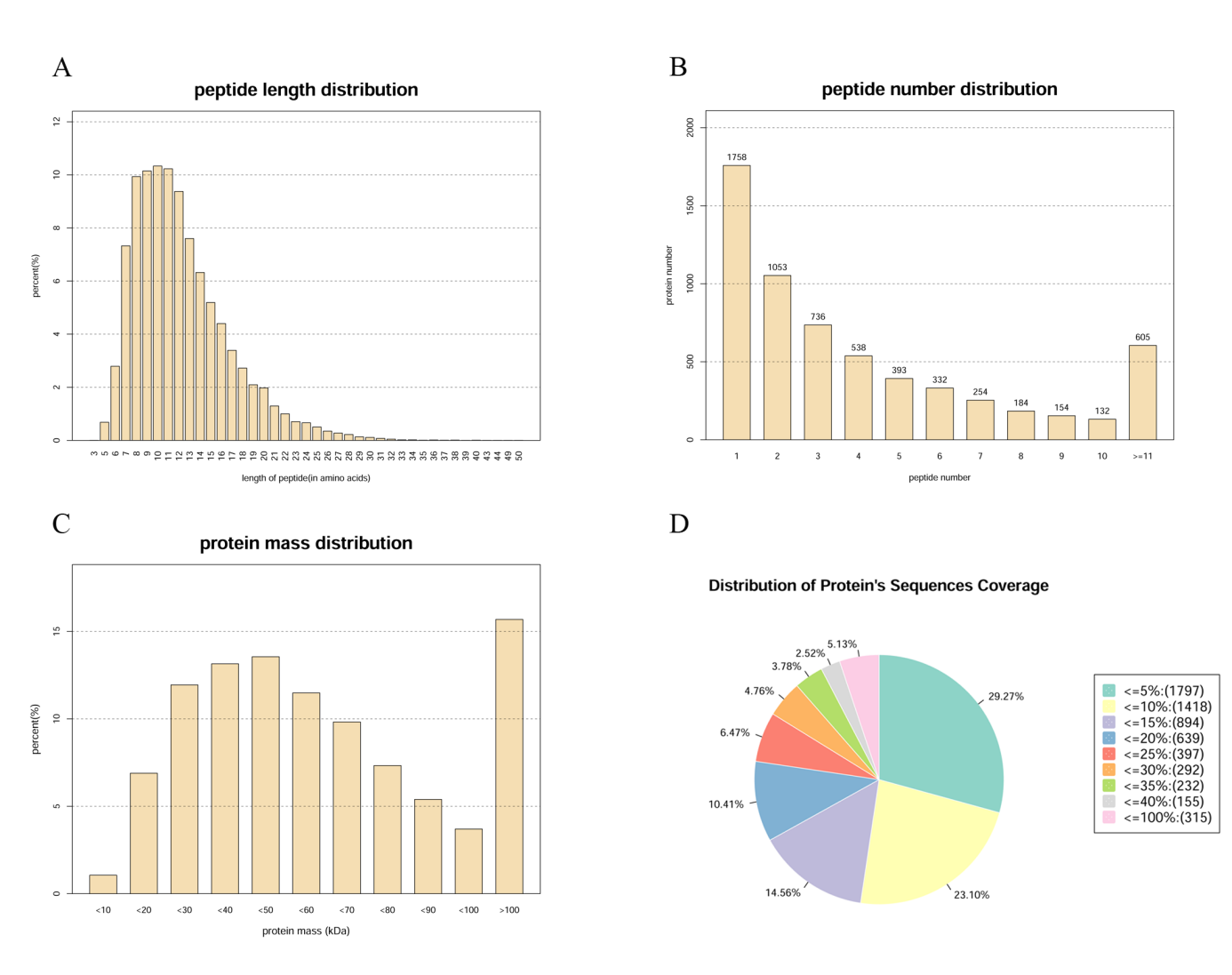


Fig S2 The distributions of the peptide length (A), number (B), mass (C) and sequence coverage (D) of the proteins of the first replicate.


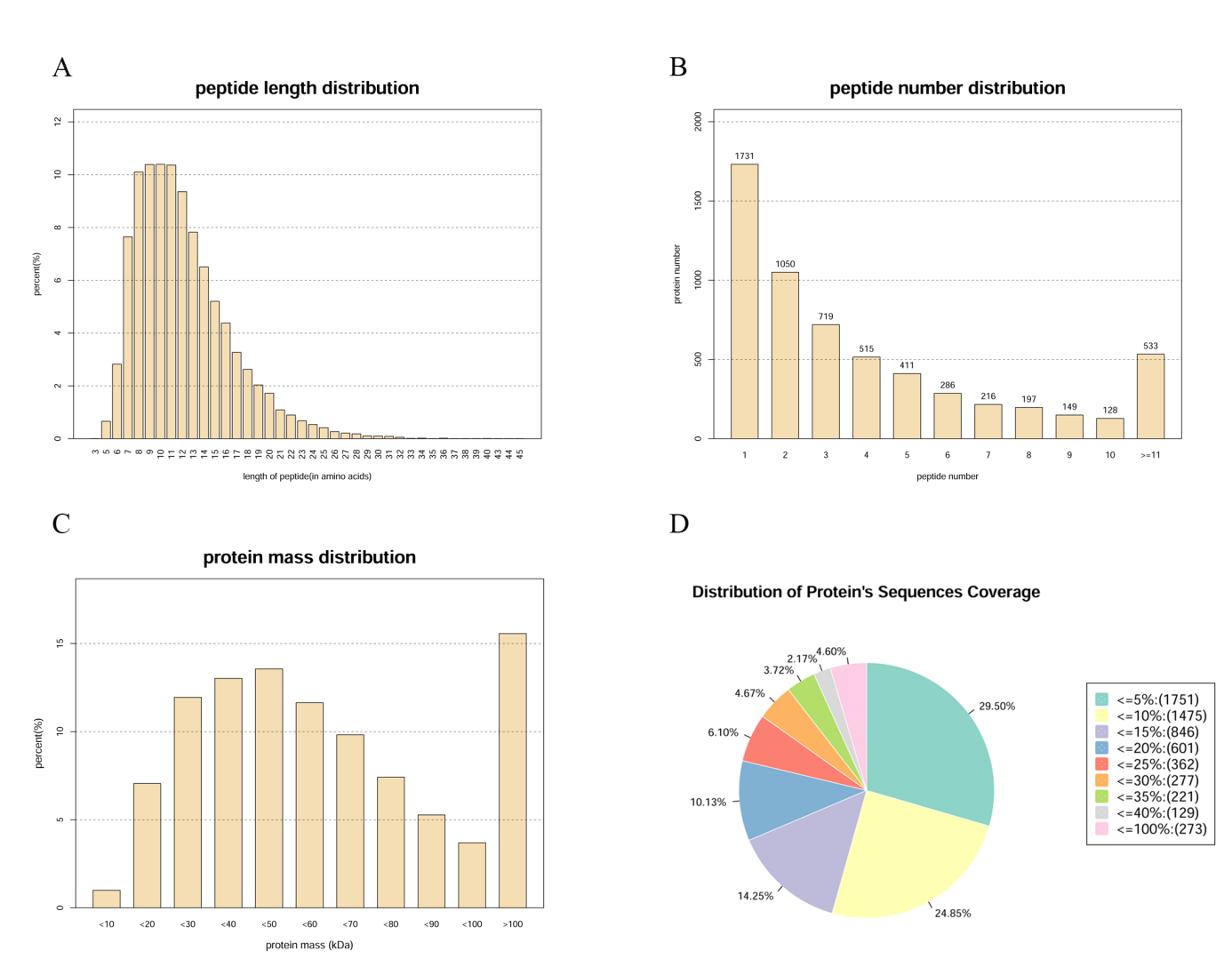


Fig S3 The distributions of the peptide length (A), number (B), mass (C) and sequence coverage (D) of the proteins of the second replicate.


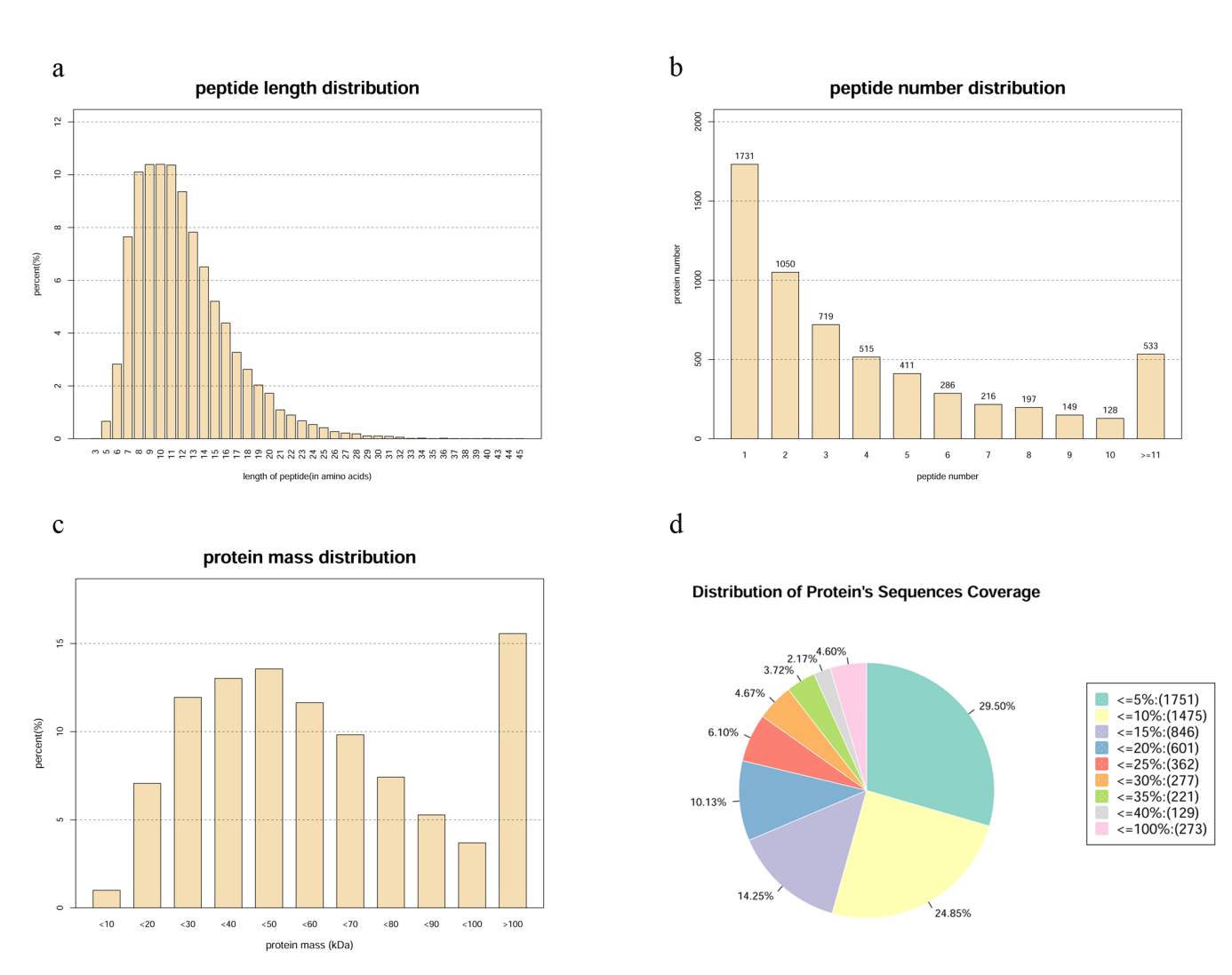


Fig S4 The distributions of the peptide length (A), number (B), mass (C) and sequence coverage (D) of the proteins of the third replicate.
